# Place of death trends among patients with dementia in Japan: a population-based observational study

**DOI:** 10.1038/s41598-019-56388-w

**Published:** 2019-12-27

**Authors:** Toshihiro Koyama, Misato Sasaki, Hideharu Hagiya, Yoshito Zamami, Tomoko Funahashi, Ayako Ohshima, Yasuhisa Tatebe, Naoko Mikami, Kazuaki Shinomiya, Yoshihisa Kitamura, Toshiaki Sendo, Shiro Hinotsu, Mitsunobu R. Kano

**Affiliations:** 1 Department of Pharmaceutical Biomedicine, Graduate School of Medicine, Dentistry, and Pharmaceutical Sciences, Okayama University, 1-1-1 Tsushima-Naka, Kita-ku, Okayama 700-8530 Japan; 2 Department of General Medicine, Graduate School of Medicine, Dentistry and Pharmaceutical Sciences, Okayama University, 2-5-1 Shikata-cho, Kita-ku, Okayama 700-8558 Japan; 3 Department of Clinical Pharmacology and Therapeutics, Tokushima University Graduate School, 3-1815 Kuramoto, Tokushima, 770-8503 Japan; 4 Department of Pharmaceutical Biomedicine, Graduate School of Interdisciplinary Science and Engineering in Health Systems, Okayama University, 1-1-1 Tsushima-naka, Kita-ku, Okayama 700-8530 Japan; 5 Department of Pharmacy, Okayama University Hospital, 2-5-1 Shikata-cho, Okayama, 700-8558 Japan; 6 Division of Pharmacy, Chiba University Hospital, 1-8-1 Inohana, Chuo-ku, Chiba, 260-8677 Japan; 7 Department of Pharmaceutical Care and Clinical Pharmacy, Faculty of Pharmaceutical Sciences, Tokushima Bunri University, 180 Nishihamabouji Yamashiro-cho, Tokushima, 770-8514 Japan; 8 Department of Biostatistics, Sapporo Medical University, South 1, West 17, Chuo-ku, Sapporo, Hokkaido, 060-8556 Japan; 9 Department of Geriatric Medicine, University of Tokyo, 7-3-1 Hongo, Bunkyo-ku, Tokyo, 113-8655 Japan

**Keywords:** Geriatrics, Health services, Epidemiology

## Abstract

Dementia is a major public health concern in ageing societies. Although the population of Japan is among the most aged worldwide, long-term trends in the place of death (PoD) among patients with dementia is unknown. In this Japanese nationwide observational study, we analysed trends in PoD using the data of patients with dementia who were aged ≥65 years and died during 1999–2016. Trends in the crude death rates and PoD frequencies were analysed using the Joinpoint regression model. Changes in these trends were assessed using the Joinpoint regression analysis in which significant change points, the annual percentage change (APC) and average APCs (AAPC) in hospitals, homes, or nursing homes were estimated. During 1999–2016, the number of deaths among patients with dementia increased from 3,235 to 23,757 (total: 182,000). A trend analysis revealed increased mortality rates, with an AAPC of 8.2% among men and 9.3% among women. Most patients with dementia died in the hospital, although the prevalence of hospital deaths decreased (AAPC: -1.0%). Moreover, the prevalence of nursing home deaths increased (AAPC: 5.6%), whereas the prevalence of home deaths decreased (AAPC: -5.8%). These findings support a reconsideration of the end-of-life care provided to patients with dementia.

## Introduction

Dementia, a progressive disorder of brain function associated with memory deficits, changes in behaviour and personality and impaired reasoning, is mainly a consequence of Alzheimer’s disease, cerebrovascular disease, and Lewy body disease. Patients with advanced dementia also tend to develop complications such as pneumonia, febrile episodes and eating problems during the final stages of life, which are associated with high mortality rates^1^. Although these complications are harbingers or direct causes of death, dementia is the major causative illness and is therefore the underlying cause of mortality reported on death certificates.

Dementia has become the leading cause of death among older people and is considered a major public health problem in the era of global ageing, given its incurable nature. The increasing rate of ageing worldwide has led to predictions that people aged ≥60 years will comprise 35% of the European population and 28% of the North American population by 2050^2^. In Japan, estimates suggest that people aged ≥65 years will comprise approximately 40% of the entire population by 2050^3^. Additional predictions suggest that between 2016 and 2050, the number of people aged ≥60 years will double in most middle-income countries, as well as in developed countries^2^. The World Health Organisation suggests that ageing profoundly exacerbates the number of patients with dementia worldwide^4^. In Japan, the Ministry of Health, Labour and Welfare publishes estimates of the populations of patients by disease and age every 3 years. According to these statistics, the estimated prevalence of dementia among people aged ≥65 years had increased from 681.9 per 100,000 people in 1999 to 2029.5 per 100,000 people in 2014^5^.

People who develop advanced dementia face a reduced survival duration, particularly due to an impaired swallowing function and complications associated with aspiration pneumonia. These issues raise concerns on the increased mortality associated with the disease^1,6^. Thus, as the number of patients with dementia has increased, end-of-life care has become a globally important subject^7^. One important indicator of the quality of end-of-life care involves whether patients can experience their last moment at a place of their choice^8^. In Japan, long-term care policies have changed over time to support the patient’s preferences regarding their end-of-life care^9^. Therefore, a deep understanding of the places of death (PoDs) of patients with dementia can provide indispensable information for planning health care policies related to the provision of end-of-life care services and the long-term planning of medical and long-term care facilities. In the United States and European countries, several studies have reported mortality rates and prevalence of PoDs among patients with dementia^10–14^. To the best of our knowledge, however, these rates remain unknown in Japan, a country with one of the most aged populations. In this study, therefore, we aimed to investigate the trends in mortality rates and PoD among older people (aged ≥65 years) with dementia in Japan by age and sex.

## Methods

### Data sources

This population-based observational study spanned an 18-year period in Japan. Data on the number of dementia-associated deaths by sex, age, and PoD were obtained from the Vital Statistics of Japan which were based on the death certificates collected by the Japanese Ministry of Health, Labour, and Welfare following the international coding rules of the World Health Organisation^15^. All death certificates are completed by a physician within 1 week after death, and the direct and underlying causes of death and PoDs are recorded and reported to the health ministry through regional health centres. Therefore, the Japanese death certificate database is a high-quality, nationally representative source of information. All deaths from 1999 to 2016 for which dementia was registered as an underlying cause of death were extracted using the International Classification of Diseases (ICD) 10^th^ revision^16^, including the codes G30 (Alzheimer’s disease), F01 (vascular dementia), and F03 (unspecified dementia) according to the previous literature^11–13^. The data of people aged ≥65 years were stratified by age and sex to determine crude mortality rates per 100,000 persons per year. To confirm the impacts of demographic changes, we calculated the age-standardised death rates per 100,000 persons to exclude the effects of demographic changes throughout the study period. Therefore, we used a direct-standardisation method based on the Japanese population structure in 1999 as a standard population and 5-year age groups (Supplementary Figure S1). The following age groups were categorised: 65–74, 75–84, and ≥85 years. PoD was defined from the death certificate and classified as hospital (hospital or physician’s office), nursing home (care home or nursing care home), or own home. The numbers of deaths by age group and place are provided in Supplementary Tables S1 and S2, respectively.

### Statistical analysis

Mortality rates are presented as crude rates per 100,000 people in the entire population and each age subgroup. The proportions of PoDs are expressed as percentages and were determined by dividing the number of deaths at each hospital, nursing home, and home by the total number of deaths occurring during the year.

To estimate trends in the mortality rate and the proportions of PoDs, the Joinpoint regression model was applied using the Joinpoint Regression Programme, version 4.6.0.0 (National Cancer Institute)^17^. The annual percentage change (APC) between trend-change points, together with its confidence interval (CI), was determined using the Joinpoint regression analysis. A *p*-value < 0.05 was defined as the significance level when the slope was statistically significantly different from zero. To compare differences in mortality trends among population subgroups, we estimated the average annual percentage change (AAPC) over the entire period. Data processing and aggregation were performed using Microsoft Access^®^ 2013 (Microsoft Corporation, Redmond, WA, USA).

### Ethics approval

This study used data provided by the Japanese Ministry of Health, Labour, and Welfare and the Statistics Bureau of the Ministry of Internal Affairs and Communications. Because these data are fully anonymised and available to the public, the ethics committee of Okayama University Hospital deemed that there was no requirement for a formal ethical review. This study was an observational study based on anonymised information, with no treatment intervention and no collection of human samples. Therefore, the requirement to obtain informed consent was waived.

## Results

**Figure 1:**
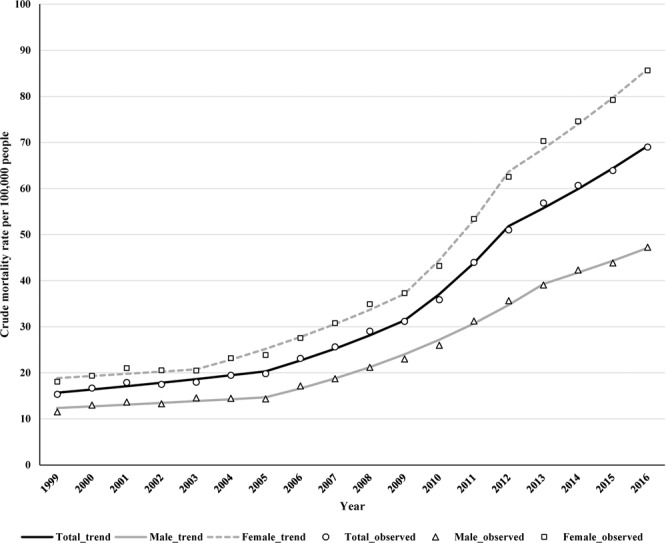
Trends in the crude mortality rates associated with dementia per 100,000 people aged ≥65 years by sex during 1999–2016.

Among older Japanese adults aged ≥65 years with dementia, 182,077 deaths attributed to dementia were recorded in the death certificate database throughout the study period. The mortality rate increased from 15.3 per 100,000 people in 1999 to 69.0 per 100,000 people in 2016 (Fig. 1).

**Figure 2:**
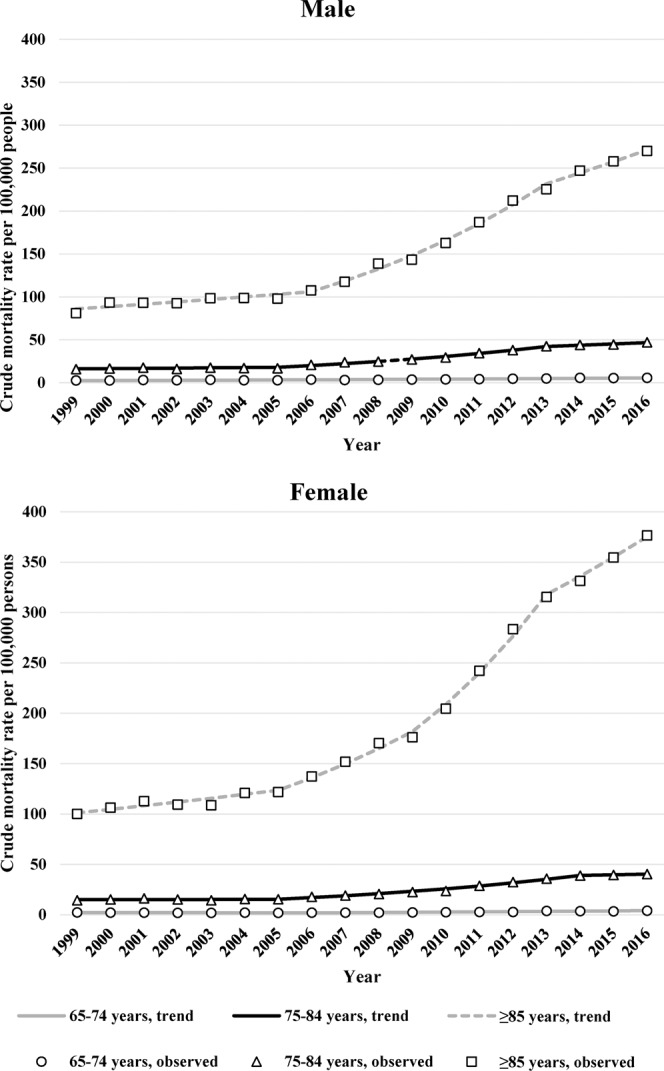
Trends in the crude mortality rates associated with dementia per 100,000 people aged ≥65 years by age during 1999–2016.

By age, the crude mortality rates per 100,000 adults with dementia aged 65–74, 75–84, and ≥85 years increased from 2.4, 14.9, and 94.6 to 4.8, 43.1, and 344.5, respectively, over the study period. The mortality rate among male adults aged ≥65 years increased from 11.5 per 100,000 people in 1999 to 47.2 per 100,000 people in 2016. The mortality rates among male adults aged 65–74, 75–84, and ≥85 years increased from 2.5, 15.8, and 81.1 per 100,000 people in 1999 to 5.7, 47.0, and 269.9 per 100,000 people in 2016, respectively (Fig. 2). Among female adults aged ≥65 years, the mortality rate increased from 18.0 per 100,000 people in 1999 to 85.6 per 100,000 people in 2016. The mortality rates among female adults aged 65–74, 75–84, and ≥85 years increased from 2.3, 14.3, and 100.1 per 100,000 people in 1999 to 4.1, 40.3, and 376.6 per 100,000 people in 2016, respectively.

**Figure 3:**
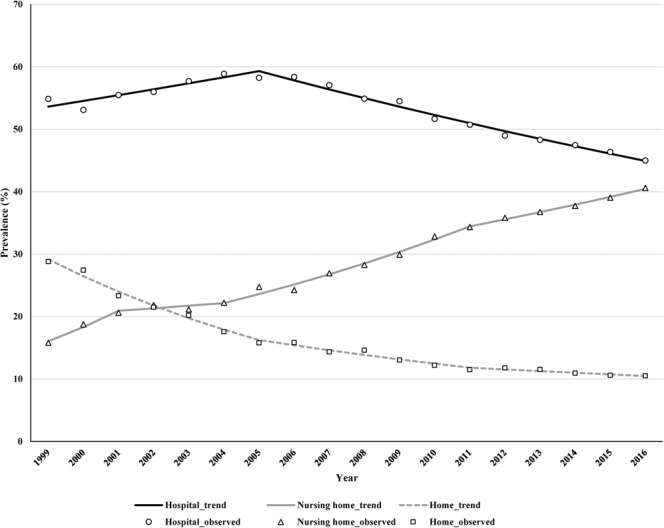
Trends in the prevalence rates of places of death among dementia patients aged ≥65 years by age during 1999–2016.

**Figure 4:**
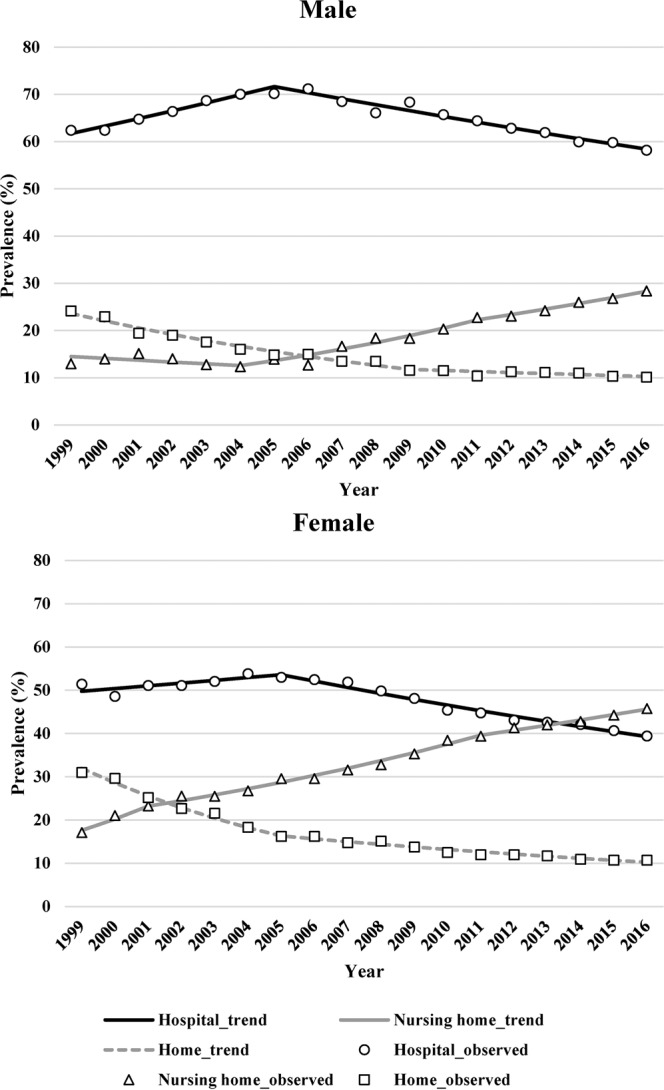
Trends in the prevalence rates of places of death among dementia patients aged ≥65 years by sex during 1999–2016.

Regarding the PoDs, the proportion of hospital deaths peaked around 2005 in both male and female adults, and declined consistently thereafter (Fig. 3). Similarly, the proportion of home deaths declined from 24.1% to 10.1% and from 30.9% to 10.7% for male and female adults, respectively (Fig. 4). In contrast, the proportion of nursing home deaths exhibited an upward trend over time, increasing from 13.0% to 28.4% in male adults and from 17.1% to 45.8% in female adults. Despite the observed decrease, hospital deaths still accounted for the highest proportion of PoDs relative to those in homes and nursing care facilities.

A trend analysis revealed that the APC of the mortality rate from dementia over the study period was 9.1% (8.2% for male and 9.3% for female adults), indicating an increase over time (Table 1). Among both male and female adults, a comparison of age subgroups revealed a higher APC of mortality among older populations.

**Table 1:** Trends in the crude death rates of patients with dementia by sex, 1999–2016.

Age group (years)	Trend 1		Trend 2		Trend 3		Trend 4		Average APC [95% CI]
	Years	APC (%)	Years	APC (%)	Years	APC (%)	Years	APC (%)	
All									
All ages	1999–2005	4.4*	2005–2009	11.5	2009–2012	18.3*	2012–2016	7.5*	9.1* [7.9 to 10.4]
65–74	1999–2007	1.0	2007–2016	7.4*					4.3* [3.1 to 5.5]
75–84	1999–2005	0.9	2005–2013	11.3*	2013–2016	4.1*	2012–2016	6.9*	6.3* [5.1 to 7.5]
≥85	1999–2005	3.2*	2005–2009	9.6*	2009–2012	16.2*			7.8* [6.7 to 8.9]
Male									
All ages	1999–2005	2.9*	2005–2013	13.1*	2013–2016	6.2*			8.2* [7.0 to 9.3]
65–74	1999–2016	5.0*							5.0* [4.2 to 5.9]
75–84	1999–2005	1.8	2005–2013	11.3*	2013–2016	3.4			6.5* [5.4 to 7.6]
≥85	1999–2006	3.0*	2006–2013	11.8*	2013–2016	5.4*			7.0* [6.0 to 8.0]
Female									
All ages	1999–2003	2.4	2003–2009	10.2*	2009–2012	19.7*	2012–2016	7.8*	9.3* [7.9 to 10.8]
65–74	1999–2007	−0.9	2007–2016	8.2*					3.8* [2.1 to 5.5]
75–84	1999–2005	0.3	2005–2014	10.9*	2014–2016	1.6	2013–2016	5.7*	6.0* [4.4 to 7.6]
≥85	1999–2005	3.4*	2005–2009	10.1*	2009–2013	15.0*			8.0* [6.9 to 9.2]

*Significantly different from zero (p < 0.05).

Abbreviations: APC, annual percentage change; CI, confidence interval.

According to the trend analysis of PoD, the proportion of hospital deaths decreased annually over the 18-year period, with an AAPC of −1.0% (95% CI, −1.3 to −0.7) (Table 2). The proportion of hospital deaths tended to increase (1.7%) until 2005, and then exhibited a downward trend (−2.5%) thereafter. The proportion of people who spent the ends of their lives in their own homes consistently decreased, with an AAPC of −5.8% (95% CI, −6.6 to −5.0). Unlike in-hospital and home deaths, the proportion of deaths in a nursing home increased consistently among both sexes, with an AAPC of 5.6% (95% CI, 3.2 to 8.0).

**Table 2:** Trends in the prevalence of places of death of patients with dementia by sex, 1999–2016.

Facility	Trend 1		Trend 2		Trend 3		Trend 4		Average APC [95% CI]	
	Years	APC [%]	Years	APC [%]	Years	APC [%]	Years	APC [%]		
**All**										
Hospital	1999–2005	1.7*	2005–2016	−2.5					−1.0*	[−1.3 to −0.7]
Nursing Home	1999–2001	14.2	2001–2004	1.9	2004–2011	6.5*	2011–2016	3.3*	5.6*	[3.2 to 8.0]
Home	1999–2005	−9.3*	2005–2011	−5.2	2011–2016	−2.4*			−5.8*	[−6.6 to −5.0]
**Male**										
Hospital	1999–2005	2.5*	2005–2016	−1.8*					−0.3	[−0.7 to 0.0]
Nursing Home	1999–2004	−2.8	2004–2011	8.5*	2011–2016	4.9*			4.0*	[2.0 to 6.1]
Home	1999–2009	−6.7*	2009–2016	−1.9*					−4.8*	[−5.5 to −4.0]
**Female**										
Hospital	1999–2005	1.2*	2005–2016	−2.8*					−1.4*	[−1.8 to −0.9]
Nursing Home	1999–2001	14.7	2001–2011	5.5*	2011–2016	2.9*			5.8*	[4.1 to 7.4]
Home	1999–2005	−10.6*	2005–2016	−4.1*					−6.5*	[−7.2 to −5.7]

*Significantly different from zero (*p* < 0.05).

Abbreviations: APC, annual percentage change; CI, confidence interval.

## Discussion

This is the first study to clarify trends in PoD among patients with dementia in Japan during 1999–2016. Notably, the mortality rates increased significantly among people with dementia aged ≥65 years throughout the study period, and tended to increase in both sexes. Given the progressive nature of population ageing, this mortality rate is expected to increase in the future. Regarding the effect of age, the increase in mortality rate between 65–74 and 75–84 years of age was higher in men than in women. This might reflect the tendency of women to have a relatively longer life expectancy^18^. This increasing mortality rate might suggest that dementia has been recognised as an underlying cause of death in Japan. Improvements in death certificate notation in the future may lead to a further increase in reported deaths attributed to dementia, which may be underreported as an underlying cause of death. Further, the number of patients with dementia in Japan also increased during this period, thus the increase in deaths related to dementia might be attributable to this increase in the number of patients with dementia.

The proportions of PoD among patients with dementia changed significantly over the study period, reflecting the ageing of Japanese society. Overall, the prevalence of hospital deaths remained high among patients with dementia, although this rate has decreased significantly since the early 2000s. The promotion of home care services by the Ministry of Health, Labour, and Welfare, along with revisions of the medical and nursing care fees since the 1980s^19^, might have contributed to the decreasing trend in hospital deaths. Regardless of government endorsements, however, the rate of dementia-associated deaths at home decreased from 28.8% in 1999 to 10.5% in 2016, with an AAPC of 5.8% over the study period. In December 2017, the Ministry of Health, Labour, and Welfare of Japan mailed a questionnaire survey regarding the preferred PoD to 6,000 randomly selected adults aged ≥20 years nationwide. The participants were instructed to assume the following: “…your dementia progresses, you need help from a caregiver for daily living, and you are considerably debilitated.” According to the results of the survey, only 3.4% of Japanese citizens wish to die of advanced dementia in a hospital, whereas 63.5% selected a nursing care facility and 32.5% preferred their own home^20^. Among people who wished to die in places other than their own homes, 76% reported not wanting to burden their family members who cared for them. The results of this study suggest a potential disparity between the hope for a particular PoD and the actual PoD. However, the long-term trends indicate a decreasing pattern in the prevalence of hospital deaths and a corresponding increase in the prevalence of nursing homes as a PoD. This suggests a narrowing gap between the ideal and reality. As the prevalence and number of dementia-associated deaths in nursing homes increases over time, readjustments to the quantity and quality of care provided in such facilities should become an important public health issue. Hospitals may provide treatment or cure for the patient’s disease and thus increase the lifespan, but may not necessarily offer care with comfort and compassion. Thus, improvements in nursing home care may be part of a major public health policy to prevent older people from dying in hospitals and improve the quality of end-of-life care in Japan. Based on this current situation, a future public health policy for patients with dementia requires serious consideration.

According to some previous studies on PoDs among patients with dementia, the prevalence of nursing homes was highest in the Netherlands at 92.3% (2003)^12^, followed by the United States at 66.9% (2001)^13^, Belgium at 65.9% (2003)^12^, the United Kingdom at 55.3% (2001–2010)^11^, and Finland at 20.5% (2013)^10^. In Japan, the prevalence of nursing homes as the choice for PoD was 30.9% in 2016. As more than 60% of Japanese individuals report a preference to die in nursing homes^20^, it may be necessary to compensate for a shortage in nursing homes for people with dementia and their families. The rate of death at home was highest in Belgium at 16.4% (2003)^12^, followed by 12.7% in the United States (2001)^13^, 8.1% (2013) in Finland^10^, 4.8% (2001–2010) in the United Kingdom^11^, and 4.7% in the Netherlands (2003)^12^. In Japan, the proportion of home deaths was 10.5% in 2016, which was consistent with previous reports from other countries. However, this rate has exhibited a downward trend and may decrease further in the future. Although these previous findings could not be compared directly because differences in the study periods and national health care systems, all suggested that people with dementia infrequently die at home. Rather, the frequency of deaths in nursing homes is increasing in many countries. However, as in-hospital deaths have decreased in Japan due to policy changes, the proportions of PoDs among patients with dementia in each of the different countries may also change in response to changing health care systems and policies. These policies should be developed and executed using limited financial resources to meet the desires of people in the respective societies.

According to a recent survey by the Economist^19^, Japan ranks 14^th^ among 80 countries worldwide for the quality of palliative care, despite its nature as an ageing society. Although a public long-term care insurance programme was established in 2000, we believe that more institutional support is required to promote end-of-life care. Moreover, advanced care planning that aims to respect an individual’s wishes should be introduced to emphasise the importance of an informed decision-making process. This study, therefore, underscores the need to consider a multidisciplinary approach to achieving patient-centred end-of-life care for dying people with dementia. This approach should be based on a deep understanding of the PoDs of patients with dementia. We should also consider improving the quality of end-of-life care to fulfil the wishes of patients not only in nursing homes.

The strengths of this study include the availability of the national death certificate database and that it is the first investigation on the long-term trends in dementia-associated mortality and PoD in Japan, one of the world’s most aged societies. Moreover, the application of the Joinpoint trend analysis to this type of research represents a very new approach which has only been reported in recent related studies^14,21^. However, the present study also has the following limitations. First, the underreporting of dementia as an underlying cause of death might have influenced the results. Second, although ICD-10 codes were utilised in this study, the validity of these data were uncertain because no clinical information on the direct association of dementia and death was available. Third, the results of the study might have been affected by long-term changes in physicians’ perceptions of dementia as a cause of death. Fourth, the PoD proportion is a relative index, as the sum of the proportions is 100%. Therefore, we provided the numbers of dementia-related deaths in each PoD in the Supplementary Table S2. Despite these limitations, this nationwide study involving an 18-year period has prominent implications for a deep understanding of the PoDs of patients with dementia and may lead to better health policy decisions for people living with dementia.

In conclusion, the dementia-associated mortality rate has increased in Japan, and the proportions of PoD have shifted between 1999 and 2016. In the near future, the majority of Japanese individuals with an underlying cause of death attributable to dementia might receive their end-of-life care in nursing homes rather than in hospitals. Therefore, the availability of nursing homes and quality of end-of-life care should be discussed among patients, families, health and nursing care providers, and policymakers to preserve the dignity of ageing individuals with dementia.

## Supplementary information

Title page and Supplementary Figure S1

Supplementary Table S1

Supplementary Table S2

## Data Availability

Data in the study is available at: https://www.e-stat.go.jp/en/stat-search/files?page=1&toukei=00450011&tstat=000001028897.
