# Supplementary material for: Place of death trends among patients with dementia in Japan: a population-based observational study
Source: Sci Rep. 2019 Dec 27;9:20235. doi: 10.1038/s41598-019-56388-w (PMC6934585; doi:10.1038/s41598-019-56388-w)

Title:

Place of death trends among patients with dementia in Japan: a population-based observational study

Author's names and affiliations

Toshihiro Koyama, PhD [1*]
Misato Sasaki, BPharm [1]
Hideharu Hagiya, MD, PhD [2]
Yoshito Zamami, PhD [3]
Tomoko Funahashi [4]
Ayako Ohshima [1]
Yasuhisa Tatebe, BPharm [5]
Naoko Mikami, BPharm [6]
Kazuaki Shinomiya, PhD [7]
Yoshihisa Kitamura, PhD [5]
Toshiaki Sendo, PhD [5]
Shiro Hinotsu, MD, PhD [8]
Mitsunobu R. Kano, MD, PhD.

[1] Department of Pharmaceutical Biomedicine, Graduate School of Medicine, Dentistry, and Pharmaceutical Sciences, Okayama University, 1-1-1 Tsushima-Naka, Kita-ku, Okayama, 700-8530, Japan
[2] Department of General Medicine, Graduate School of Medicine, Dentistry and Pharmaceutical Sciences, Okayama University 2-5-1 Shikata-cho, Kitaku, Okayama, 700-8558, Japan
[3] Department of Clinical Pharmacology and Therapeutics, Tokushima University Graduate School, 3-1815 Kuramoto, Tokushima, 770-8503, Japan
[4] Department of Pharmaceutical Biomedicine, Graduate School of Interdisciplinary Science and Engineering in Health Systems, Okayama University, 1-1-1 Tsushima-naka, Kita-ku, Okayama, 700-8530, Japan
[5] Department of Pharmacy, Okayama University Hospital, 2-5-1 Shikata-Cho, Okayama 700-8558, Japan
[6] Division of Pharmacy, Chiba University Hospital, 1-8-1 Inohana, Chuo-Ku, Chiba 260-8677, Japan
[7] Department of Pharmaceutical Care and Clinical Pharmacy, Faculty of Pharmaceutical Sciences, Tokushima Bunri University. 180 Nishihamabouji Yamashiro-cho, Tokushima

770-8514, Japan
[8] Department of Biostatistics, Sapporo Medical University, South 1, West 17, Chuo-Ku, Sapporo, Hokkaido 060-8556, Japan
[9] Department of Geriatric Medicine, University of Tokyo, 7-3-1 Hongo, Bunkyo-Ku, Tokyo 113-8655, Japan

Corresponding Author:
Toshihiro Koyama, PhD
Department of Pharmaceutical Biomedicine, Graduate School of Medicine, Dentistry, and Pharmaceutical Sciences, Okayama University 1-1-1 Tsushima-Naka, Kita-ku, Okayama, 700-8530, Japan
Tel.: +81-86-235-6585
E-mail: koyam-oka@umin.ac.jp

Supplementary materials

Supplementary Figure S1. Trends in age-standardized mortality rate from dementia per 100,000 people by sex, 1999-2016

Supplementary Table S1. Number and crude rate of death from dementia per 100,000 people by age group, 1999–2016

Supplementary Table S2. Number, percent, and annual percent change of death from dementia, 1999–2016

Supplementary Figure S1. Trends in age-standardized mortality rate from dementia per 100,000 people by sex in 1999-2016

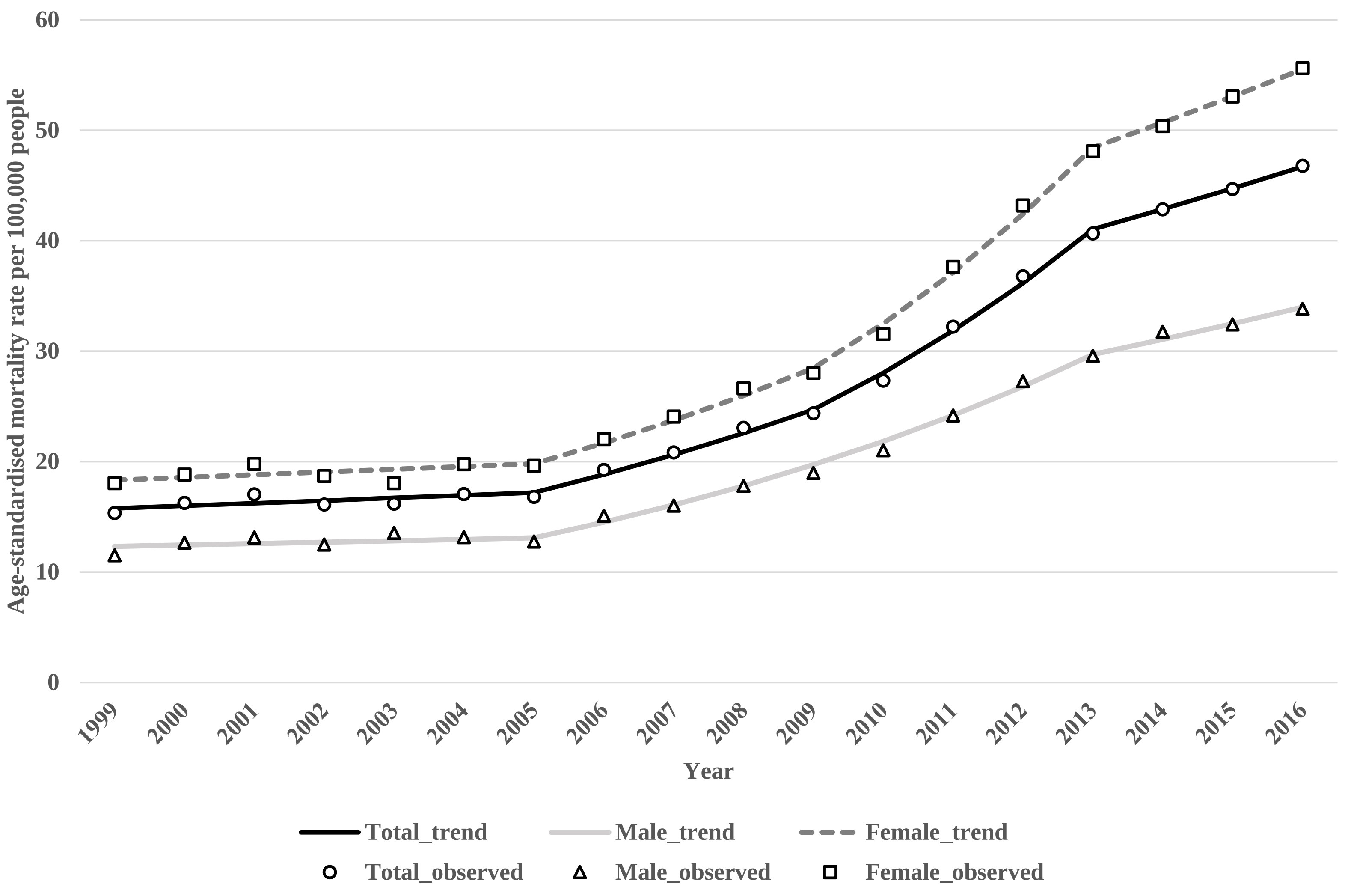

Supplement: Supplementary file 1 — Title page and Supplementary Figure S1 [file 41598_2019_56388_MOESM1_ESM.pdf]
